# Clinic of the Jefferson Medical College

**Published:** 1844-02-10

**Authors:** 


### CLINICAL LECTURES AND REPORTS.

### PHILADELPHIA HOSPITAL.

**CLINIC OF THE JEFFERSON MEDICAL COLLEGE.**

*January* 20, 1844.

BY PROFESSOR DUNGLISON.

(Reported by S. G. White, and E. R. Squibb.)

#### INTERMITTENT FEVER.

The Professor commenced by remarking, that there were several cases in the wards, which had been arrested by the sulphate of quinia, but in which the cure was not permanent. Also, some which had altogether resisted the use of the sulphate. A majority of these cases were quotidians; some double quotidians. In these obstinate cases he had prescribed, along with the sulphate of quinia, the use of the liquor arsenicalis, "Fowler's Solution," the administration of which had been attended with great success. Five cases of intermittent were described, some of which had yielded to the ordinary treatment; others which had required the use of arsenic to render the cure permanent. One of the cases illustrative of the benefit resulting from this treatment was the following:

Ann F., came into the hospital with remittent fever, for which she was treated in the usual manner, and recovered: soon afterwards she was attacked with intermittent, which yielded temporarily to the sulphate of quinia. The disease recurred, and was removed by arsenic, which in her case induced puffiness of the face, when it was discontinued. About two weeks ago, she complained of excessive pain between the crista ilii and the cartilages of the ribs of the left side, which was relieved by cups and leeches, with warm fomentations, so that she was able to walk about the ward. The pain soon returned, however, preceded by a rigor. Like the intermittent itself, it appeared to be neuropathic, and was relieved at once by a large injection with Oleum Terebinthinæ. After this she was again seized with intermittent fever, which was finally subdued by the use of sulphate of quinia and arsenic; since which she has been convalescent. The Professor advises first the exhibition of the sulphate of quinia, or the bark in substance; and should these fail, arsenic may be resorted to in conjunction, and generally with full success. He then proceeded to make some remarks upon

#### CHRONIC DIARRHŒA AND DYSENTERY,

Dependent upon ulceration of the mucous lining of the intestinal canal. In ordinary diarrhœa, gentle cathertics, as the Oleum Ricini, used daily to remove morbid secretions, and warmth to the surface, with easily digested food, are sufficient for a cure. Cases, however, not unfrequently occur, which resist all ordinary methods of treatment, and several such are now in the wards. Astringents are always resorted to, but in most cases they seem to act mainly by their influence on the stomach. They can scarcely be expected to exert much direct effect on the parts affected, especially when they are seated low down in the canal. All of them must become mixed with the contents of the canal; and be in a state of extreme dilution before they can come in contact with the diseased surface. The lecturer instanced particularly the nitrate of silver, which is often prescribed in not more than quarter or half grain doses, under the impression that it may act locally on the ulcerations. This salt, he conceived, could never pass the stomach unchanged. It must be converted in the stomach into a chloride, to which the good effects must be referred, should any ensue. If so, then why not give the chloride? This had been done, and he was inclined to believe it possessed all the powers of the nitrate. Dr. Perry, of Philadelphia, whilst resident Physician of the Hospital, had often administered it, and he thought with advantage. The lecturer refered to obstinate cases of ulceration, which had resisted the laxative, astringent, terebinthinate, mercurial and external treatment, and stated, that they must be left to the recuperative powers, under which they may cicatrize; but too often they wear out the patient gradually, or terminate in perforation and death, of which the class had seen an example lately.

#### PNEUMONITIC ABSCESS.

The Professor took occasion to mention, that the case of pleuro-pneumonia terminating in pulmonic abscess, which he had exhibited at a previous lecture, was gradually failing, the abscess having invaded the lung almost to its apex; that marked hectic had supervened, and he did not think the patient could survive many days. He expected to have to direct their attention to the case again at an early period.

#### CARDIAC DISEASE.

An extraordinary and interesting case was now presented before the class, in which the diagnosis was by no means easily made out. The patient, a sailor, had suffered five years ago from acute rheumatism, from which he recovered and remained well until about eighteen months since, when he was in the hospital under the lecturer's care, laboring under dropsy dependent upon valvular disease of the heart; the consequence, probably, of endocarditis in the former attack of acute rheumatism. He was treated for these affections, which appeared to have mainly yielded, with the exception of more or less dyspnœa on making exertion; when he was seized with the initial symptoms of his present condition. These at present are, pain over the spine between the shoulders; tenderness on pressing the parietes of the chest anteriorly; some dysphagia—at times partial, at others throughout the tube; respiration very variable; sometimes as high as sixty-four per minute, and blueness of face and lips. About three months since, he perceived a tumour between the cartilages of the third and fourth ribs, at the left margin of the sternum, which has gradually developed itself, and has either pushed aside, or partially absorbed, the cartilage of the fourth rib. The whole præcordial region is prominent, and heaves with every beat of the heart. When the finger presses on the tumour, fluid is plainly perceptible, and at each systole of the heart, the finger is elevated. The spiral turn of the heart from left to right, during the systole is distinctly seen. Pulse, under excitement, small and soft; ordinarily, hard and tense; a withdrawal of the apex of the heart from the region below the nipple at every pulsation; no venous pulse; percussion from third rib to the base of the chest dull, and very painful; a thrill is felt in the prominence during the diastole, but none during the systole; second sound is harsh and loud, louder the lecturer thought than any valvular sound he had ever heard, like whispered *hoo*, most distinct over the tumour, or near the apex of the heart. Both sounds are modified, the first being confused and heard distinctly. Posteriorly, they are feeble and distant. Has had considerable œdema of the limbs, which still exists to a slight degree. The difficulties in the diagnosis were described by the Professor. The pulsation in the tumour, together with the functional phenomena and certain of the physical signs might lead to the conclusion that aneurism exists. The tumour appears to be too low down for aneurism of the ascending aorta, and the phenomena would scarcely indicate the descending. It may possibly be an abscess of the chest, pulsation being communicated to it by the heart; but along with this there must be serious disease of the valves as indicated by the rasping sound during the diastole.

The Professor drew upon the black board a representation of the heart seen in section, and explained the two sounds of the heart in health and disease, and their connection with the systole and diastole of the organ, and inferred, that the abnormous second sound occurring during the diastole might be referred to a morbid state of the semilunar valves of the aorta; yet the sound was heard more distinctly over the tumour, or near the apex, than immediately over the valves.

After a careful and minute investigation of all the phenomena, he was disposed to lean to the belief in the existence of aneurism, yet it was difficult to say where it was seated; and that it is possibly complicated with an abscess anterior to the pericardium. If such be the nature of the case, no therapeutic treatment is likely to prove beneficial; he advised perfect rest of body and mind. Had the patient been of active habit it would have been well to prevent the accumulation of too much blood. In this case, however, this course would not be necessary or proper, as the patient is rather anæmic. To throw light on the case, we must wait the further development of phenomena, which the Professor stated he would report to the class.

### ANEURISM OF THE AORTA, &c.

An interesting pathological specimen of aortic aneurism, which had not been suspected during the life of the patient, was exhibited.

The patient, a colored female, aged 34, entered the house, presenting marked symptoms of bronchitis—fever, cough, considerable dyspnœa, free mucous expectoration, which was colourless and frothy; percussion clear; rhonchi of almost every description continually changing from one to another; indeed, every symptom indicating extensive bronchitis of both lungs. She was treated by blisters, remedies to relieve the cough, &c., which afforded but temporary relief. The first sound of the heart was roughened (bellows' sound,) the second clear and distinct, and the action of the organ irregular, and spasmodic, as it were; but no sounds were heard which led to the suspicion of aneurism. Two days before her death, she was attacked with evident signs of pneumonia of the lower lobe of the left lung, and under these complications she sank.

Dissection showed an aneurism of the arch of the aorta, the sac of which was almost filled with fibrinous matter. The lining membrane of the aorta was much inflamed and injected,—(*endo-aortitis.*) The heart was natural with the exception of the mirtal valve, which was wasted and therefore insufficient. This accounted for the bellows' sound accompanying the first sound of the heart, and was doubtless owing to regurgitation through the auriculo-ventricular opening. The lower lobe of the left lung exhibited the first stage of pneumonitic condensation. The corresponding portion of the pleura pulmonalis was likewise inflamed.

The Professor concluded by some remarks in the great difficulty that occasionally exists in the diagnosis of aneurism of the aorta,—so great, that excellent observers have concluded there is no pathognomonic sign. He referred to three published cases, which had occurred to gentlemen of eminence, and were treated as cases of chronic laryngitis,—the rhonchi, affection of the voice, and other morbid phenomena, referable to the air passages, being produced by the pressure of the aneurismal tumour. He had no doubt that in the case of this female, many of the anomolous rhonchi were caused in this manner, as well as the bronchitis itself.

